# Excess Success for Psychology Articles in the Journal *Science*

**DOI:** 10.1371/journal.pone.0114255

**Published:** 2014-12-04

**Authors:** Gregory Francis, Jay Tanzman, William J. Matthews

**Affiliations:** 1 Department of Psychological Sciences, Purdue University, West Lafayette, Indiana, United States of America and Brain Mind Institute, École Polytechnique Fédérale de Lausanne, Lausanne, Switzerland; 2 Tanzman Statistical Consulting, Los Angeles, California, United States of America; 3 Department of Psychology, University of Cambridge, Cambridge, United Kingdom; The Ohio State University, Center for Cognitive and Brain Sciences, Center for Cognitive and Behavioral Brain Imaging, United States of America

## Abstract

This article describes a systematic analysis of the relationship between empirical data and theoretical conclusions for a set of experimental psychology articles published in the journal *Science* between 2005–2012. When the success rate of a set of empirical studies is much higher than would be expected relative to the experiments' reported effects and sample sizes, it suggests that null findings have been suppressed, that the experiments or analyses were inappropriate, or that the theory does not properly follow from the data. The analyses herein indicate such excess success for 83% (15 out of 18) of the articles in *Science* that report four or more studies and contain sufficient information for the analysis. This result suggests a systematic pattern of excess success among psychology articles in the journal *Science*.

## Introduction

Unbelievable discoveries [1], important experimental findings that fail to replicate [2], [3], fraudulent data [4], [5], and awareness that researchers might use questionable research practices to produce significant findings [6], [7] have contributed to concerns that psychology cannot be trusted to produce valid scientific work [8]–[10]. Even though fraud may be rare, researchers may re-run experiments, drop subjects, selectively merge data from different experiments, suppress null findings, drop experimental conditions, or round down *p* values in order to report statistical significance. Such practices bias the experimental outcomes, undermine the credibility of the theoretical conclusions that are derived from the published experimental data, and leave a statistical trace that can be identified across multiple experiments with the Test for Excess Significance (TES) [11]. Broadly speaking, the TES estimates the probability that a set of experiments with proper sampling, appropriate analyses, and full reporting will produce at least as many “successful” outcomes as have actually been observed. If this probability is small, it suggests that researchers should doubt the assumptions of appropriate sampling, proper analysis, and complete reporting. We describe the details of the TES below.

Recent investigations using the TES [12]–[21] have indicated that some articles and meta-analyses in the field of psychological science appear to be biased, which suggests that scientists should be skeptical about the claims in those articles and meta-analyses. Since those TES analyses focused on specific articles rather than a representative sample of articles, they do not allow for generalization to the broader field. It is difficult to create a random sample from published articles, and such a sample may not be especially meaningful because a seminal paper may motivate many investigations while a randomly selected paper may have little impact. Francis [22] partly addressed this issue by investigating all possible articles in the prominent journal *Psychological Science* over a four-year span. In that analysis, 82% of investigated articles (36 out of 44) failed the TES analysis. Here we apply the same systematic analysis to articles published in the highly influential journal *Science* over an eight-year span. We investigated the journal *Science* because it is widely recognized as being one of the most important scientific journals. We restricted our analysis to papers related to psychology and education because, as described below, the TES analysis requires some subject-matter expertise to be able to interpret the presented statistical findings. The current authors have such expertise for psychology and education but will have to leave a similar analysis for fields such as biology or medicine to other subject-matter experts.

## The Test for Excess Success

Across a series of hypothesis tests some unknown subset of the tests will result in errors, either by rejecting the null hypothesis when it is true (a Type I error) or by failing to reject the null when it is false (a Type II error). Random sampling ensures that such errors will sometimes occur even under ideal experimental conditions. Null hypothesis significance testing provides a way to control the Type I and Type II error rates; for example, setting the Type I error rate at 0.05 implies that one will mistakenly reject the null hypothesis for 5% of those studies where the null hypothesis is true. Under non-ideal conditions, such as when model assumptions required by the statistical tests are not met, the Type I error rate can be much larger than the intended 5%. Moreover, this nominal error rate depends upon the data having been properly sampled, analyzed, and reported.

Improper sampling, analysis, or reporting is difficult to identify in any single study, but such behaviors leave a detectable pattern across a set of experiments. Ioannidis and Trikalinos [11] proposed a “test for excess significance” (TES) that compares estimates of experimental power with the reported frequency of significant outcomes. For our analyses, we slightly extend the TES to encompass “excess success” rather than only excess significance.

The definition of “success” differs across experiments. In many experiments a successful outcome is to reject the null hypothesis (typically defined as *p≤*0.05), in which case the probability of success corresponds to a calculation of experimental power. In other experiments a successful outcome is to not reject the null hypothesis, in which case the success probability is the complement of power. Finally, an experiment's success is sometimes based on a pattern of significant and non-significant hypothesis tests that contrast different aspects of the data. Regardless of the definition of success, a set of experiments with properly gathered data, and results that are appropriately analyzed and fully reported, should produce successful outcomes at a rate consistent with the experiments' estimated success probabilities [11], [15], [18], [21]. Too much success suggests that the reported results are biased in favor of the theoretical claims.

The logic of the TES is similar to standard approaches in hypothesis testing. We start by supposing proper data collection and analysis for each experiment along with full reporting of all experimental outcomes related to the theoretical ideas. Such suppositions are similar to the null hypothesis in standard hypothesis testing. We then identify the magnitude of the reported effects and estimate the probability of success for experiments like those reported. Finally, we compute a joint success probability, *P_TES_*, across the full set of experiments, which estimates the probability that experiments like the ones reported would produce outcomes at least as successful as those actually reported. When the reported experiments are uniformly successful, *P_TES_* estimates the probability that direct replications of the experiments will all be successful. The *P_TES_* value plays a role similar to the *p* value in standard hypothesis testing, with a small *P_TES_* suggesting that the starting suppositions are not entirely correct and that, instead, there appears to be a problem with data collection, analysis, or publication of relevant findings. In essence, if *P_TES_* is small, then the published findings in an article appear to be “too good to be true” relative to the theoretical claims. A common criterion for *P_TES_* being small is 0.1 [11], [12], [23]. Given scientific interest in reproducibility, a probability of 0.1 seems like a very modest criterion for success across experiments that support theoretical claims; most scientists would probably want their theoretical claims to be based on experiments with much more reliable outcomes. While it is not the case that the theoretical claims derived from experiments with excess success are necessarily (or entirely) wrong, the evidence for these claims is, at best, weaker than it first appears.

To explain the TES analysis without emphasizing any particular article, Table 1 describes the relevant statistics and hypotheses for five reported experiments that are taken from five different articles in *Science* and artificially brought together. The TES conclusion here will not be meaningful because these experiments do not promote a common theoretical claim, but the discussion helps to demonstrate the types of issues that appear when doing a TES analysis. The original authors reported the hypotheses listed in Table 1 as supporting their theoretical claims. Because “success” sometimes includes a complex set of both significant and non-significant outcomes, the estimation of success probability is often more complicated than a standard power analysis. Moreover, sometimes an article does not report sufficient statistical detail to fully estimate the success probabilities. In such cases, we always estimate an upper limit on the probabilities, which favors an interpretation that the articles are valid.

**Table 1:** Statistical properties, hypotheses, and estimated probabilities of success for a set of five experiments.

	Statistics	Hypotheses	Probability of success
Exp. 1	*n* = 179	ρ_1_ ≠ 0	0.844
	*r_1_* = −0.22	ρ_2_ ≠ 0	0.518
	*r_2_* = −0.15	ρ_3_ ≠ 0	0.675
	*r_3_* = −0.18	Joint	*0.518*
Exp. 2	*n_1_* = 17, *n_2_* = 17, *n_3_* = 18	ANOVA	0.684
	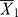 = 316, 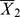 = 305, 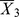 = 186	µ_1_ ≠ µ_3_	0.696
	*s* = 152	µ_2_ ≠ µ_3_	0.620
		µ_1_ = µ_2_	0.946
		Joint	*0.482*
Exp. 3	*n_1_* = 18, *n_2_* = 18		
	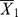 = 5.16, 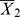 = 3.47	µ_1_ ≠ µ_2_	*0.517*
	*s* = 2.85		
Exp. 4	*n_1_* = 28, *n_2_* = 26	µ_X1_ ≠ µ_X2_	0.495
	*F_X_ = 4.08, F_Y_ = 4.40*	µ_Y1_ ≠ µ_Y2_	0.528
	*r_XY_* = 0.36	Joint	*0.318*
Exp. 5	*n_1A_* = 17, *n_2A_* = 17, *n_1B_* = 17, *n_2B_* = 17	Interaction	0.916
	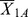 = 3.87, 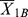 = 7.00	µ_1A_ ≠ µ_1B_	0.681
	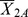 = 7.59, 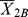 = 4.28	µ_2A_ ≠ µ_2B_	0.635
	*s* = 3.91	Joint	*0.438*
*P_TES_*			0.018

Experiment 1 measured the correlation between a dependent variable and each of three related measures of another variable. The probability of a significant outcome for each test by itself is estimated with post hoc power calculations [24], which suppose that the population correlation is the value observed in the sample. (Post hoc power is sometimes inappropriately used as an estimated probability of an already observed experimental outcome, but we use it here as an estimate of success probabilities for the outcome of replication studies.) Because these outcomes are correlated, estimating the joint probability for all three observed significant outcomes would require access to the raw data. Since the probability of all three outcomes being significant must be less than the probability of any one of the outcomes, the test with the smallest success probability provides an estimated upper bound for the set. This estimated probability is a bit over one-half, and Table 1 lists it as the joint probability for Experiment 1.

Experiment 2 used a between-subjects design with three conditions. The authors supported their theoretical claims with a statistical analysis that included a significant omnibus ANOVA and significant contrasts between a control and each of the two experimental conditions. Any difference between the two experimental conditions was predicted to be non-significant. These tests are not independent, so we estimated the probability of success by the Monte Carlo method. We simulated 100,000 experiments that drew samples from normally distributed populations using the means and standard deviations derived from the sample statistics. Our simulations found that only the predicted non-significant outcome has a high probability of success. Moreover, the joint probability of all tests producing a successful outcome is less than one-half because it is uncommon for a set of random samples to satisfy so many constraints on the outcomes.

Experiment 3 compared ratings across two priming conditions with a two-sample *t*-test. The test produced only a “marginally significant” result (*p* = 0.09), but this was judged by the original authors as sufficient evidence to support their theoretical claim. To be consistent with the original authors, the success probability was based on a significance criterion of 0.1.

Experiment 4 reported two behavioral measures from two samples of participants that were exposed to different conditions. A successful outcome relative to the theory required both measures to show a significant difference. The summary statistics did not fully report the means and standard deviations of the measures, so Table 1 lists the relevant *F* values for the tests. The article reported the correlation between the measures, so we estimated the probability of success by the Monte Carlo method with 100,000 simulated experiments that took samples from populations using the correlation and standardized means, which we derived from the *F* values. The success probability for each individual test is close to one-half, but the probability of both outcomes being significant is closer to one-third. Similar to Experiment 2, the multiple constraints on the definition of success reduce the joint probability.

Experiment 5 used a two-by-two between-subjects design and predicted a significant interaction and significant contrasts across each of two pairs of conditions. We estimated the probabilities of these outcomes with simulated experiments that used the reported means and estimated standard deviations. We derived the latter from the reported *F* values because the reported standard deviations were inconsistent with the reported *F* values. Although the interaction has a high success probability, the joint success probability is low because the particular type of interaction required by the theoretical claims is fairly uncommon.

The italicized success probability for each experiment in Table 1 indicates the joint probability for all of the required outcomes for that experiment. The estimated probability that five experiments like these would all produce successful outcomes is the product of the five joint probabilities, *P_TES_* = 0.018. This probability indicates that entirely successful outcomes across all of these experiments should be very rare for unbiased experiments. Had these experiments been reported together, in a single paper, to support a set of related theoretical claims, then such a low probability would indicate that readers should be skeptical that the data were gathered properly, that the analyses were appropriate, or that all relevant experimental findings were fully reported. Note that this skepticism would not mean that the theoretical claims were wrong, only that such claims were unsubstantiated by the analyses of the current set of experiments. This skepticism also would not necessarily indicate that any of the experimental results were invalid, because the TES analysis can only identify a problem across the set. It could be that all of the reported experiments were valid but that unsuccessful valid experiments were not reported. Such unsuccessful experiments might undermine the authors' theoretical claims. Alternatively, the reported experiments might be invalid by themselves because of inappropriate sampling or analysis.

## Applying the TES Analysis to Articles in *Science*

From the *Science* journal's on-line collection, we downloaded all 133 original research articles (and their supplementary material) that were classified as Psychology or Education for years 2005–2012. We then checked each article and its supplementary material to determine if the article contained four or more studies, a condition required to provide sufficient power to conduct a TES analysis [18]. We identified 25 such articles classified as Psychology and one such article classified as Education.

We further examined each of these 26 articles to see if the article and its supplementary material provided sufficient detail to perform a TES analysis. Eight articles did not include sufficient detail to compute success probabilities for at least four studies and were thus excluded from the TES analysis. Information S1 lists the eight excluded articles and the reasons for their exclusion. The supporting information also provides a full description of the TES analysis for each of the included articles [25]–[42] (Information S1) and provides *R* source code (Information S2) for estimating success probabilities with Monte Carlo simulations for complicated experimental designs.

## Results and Discussion

Table 2 lists the *P_TES_* value for each of the 18 analyzed articles. For 15 of these articles, the probability of the observed experimental outcome is below the 0.1 criterion for excess success. Thus, 83% of the articles in Table 2 make theoretical claims based on experimental results that appear to be too good to be true given a weak requirement for estimated reproducibility. This 83% apparent bias rate is especially troubling because the journal *Science* publishes studies that are widely considered to be among the best and most influential in the field. If the work published in *Science* is flawed, then either the entire field is suspect or the journal *Science* does not actually reflect the field's best work and its influence is unwarranted.

**Table 2:** Results of the TES analysis for each of eighteen articles in *Science*.

Year	Authors	Short title	*P_TES_*
2006	Dijksterhuis et al. [25]	Deliberation-Without-Attention Effect	0.051
2006	Vohs et al. [26]	Psychological Consequences of Money	0.002
2006	Zhong & Lijenquist [27]	Washing Away Your Sins	0.095
2007	Wood et al. [28]	Perception of Goal-Directed Action in Primates	0.031
2008	Whitson & Galinsky [29]	Lacking Control Increases Illusory Pattern Perception	0.008
2009	Mehta & Zhu [30]	Effect of Color on Cognitive Performance	0.002
2009	Paukner et al. [31]	Monkeys Display Affiliation Toward Imitators	0.037
2009	Weisbuch et al. [32]	Race Bias via Televised Nonverbal Behavior	0.027
2010	Ackerman et al. [33]	Incidental Haptic Sensations Influence Decisions	0.017
2010	Bahrami et al. [34]	Optimally Interacting Minds	0.332
2010	Kovács et al. [35]	Susceptibility to Others' Beliefs in Infants and Adults	0.021
2010	Morewedge et al. [36]	Imagined Consumption Reduces Actual Consumption	0.012
2011	Halperine et al. [37]	Promoting the Middle East Peace Process	0.210
2011	Ramirez & Beilock [38]	Writing About Worries Boosts Exam Performance	0.059
2011	Stapel & Lindenberg [39]	Disordered Contexts Promote Stereotyping	0.075
2012	Gervais & Norenzayan [40]	Analytic Thinking Promotes Religious Disbelief	0.051
2012	Seeley et al. [41]	Stop Signals Provide Inhibition in Honeybee Swarms	0.957
2012	Shah et al. [42]	Some Consequences of Having Too Little	0.091

It remains an open question whether the 83% excess success rate generalizes to studies in *Scienc*e with fewer than four studies. On the one hand, papers with fewer studies may be based on more convincing experimental results (e.g., larger sample sizes), which might indicate that the 83% rate should not apply to such papers. On the other hand, it seems unfair to suppose that scientists would lower their standards for papers with four or more experiments, which suggests that the same problems that produce the 83% rate would also apply to other papers. Another open question, to which similar considerations apply, is whether the excess success rate for *Scienc*e generalizes to other journals. It could be that journals that impose different publication criteria than *Scienc*e end up publishing more convincing experimental results, but it would be ironic if scientists' most valid work was published in “secondary” outlets. Furthermore, the excess success rate in *Science* is similar to the reported excess success rate in *Psychological Science* (82%), where the only other systematic TES analysis of psychology articles has been applied [22].

Two of the articles listed in Table 2 deserve special discussion. One of the articles has been retracted due to Stapel's fraudulent research practices [4], [39]. The TES analysis is not designed to detect fraudulent data, because a knowledgeable fraudster can always craft data that pass the test and produce a seemingly convincing scientific argument. Although Stapel remains responsible for his fraud, knowledgeable researchers in the field could have identified that his reported findings were too good to be true (*P_TES_* = 0.075). Likewise, when evidence of fraud was levied against Marc Hauser in other publications, his article in *Science* [28] was suspected of improper data collection. The article was subsequently “cleared” by a replication experiment, but the originally published data seem too good to be true (*P_TES_* = 0.051), and the subsequent successful replication made the full set of findings even less believable (*P_TES_* = 0.031).

Although many of the concerns about research practices have focused on articles from social psychology, Table 2 demonstrates that some studies from educational psychology [38], developmental psychology [35], and primate behavior [28], [31] have similar problems.

The bias across the studies in Table 2 must be severe because simulation studies of the performance of the TES analysis [15], [18] demonstrate that the test is conservative, in the sense that truly proper experiment sets rarely produce *P_TES_* values below the 0.1 criterion. When a set of unbiased experiments all happen to produce a significant effect, such experiments also tend to give large estimated power values and thereby produce a large *P_TES_* value. If the true power is small, it is unusual for all of the experiments to produce a significant outcome, but it is even more unusual for such experiments to have small estimated power values. For unbiased experiment sets, the true Type I error rate for concluding bias (reporting bias that does not exist) is often close to 0.01 even when using the nominal 0.1 criterion. Furthermore, when only a file-drawer bias exists (running proper experiments but suppressing unsuccessful findings), the test often fails to detect the bias because inflated effect size estimates from the biased set of published experiments lead to overestimated success probabilities. Thus, the high rate of bias detected in Table 2 is unlikely to be produced only by the suppression of null findings. Instead, it seems that multiple forms of bias were applied to the articles in *Science*. It seems plausible that researchers often tweak their datasets or analyses to produce *p* values just below 0.05 and also reinterpret or suppress findings when they produce *p* values that cannot be forced below the significance criterion [43].

## Problems with Scientific Practice in Psychology

The TES analyses in Table 2 paint a worrying picture of the psychology research that is published in the journal *Science*. However, as noted previously, this does not necessarily imply that the authors of apparently biased articles intentionally misled readers. This section discusses how four scientific principles can be easily misapplied and how those misapplications tend to produce experimental results with excess success. This discussion is not intended to be exhaustive or entirely novel, but it tries to focus on issues that might explain the findings in Table 2.

### Replication Does Not Necessarily Establish Scientific Truth

Successful replication is widely considered to be the gold standard of empirical scientific investigations. However, the role of replication in a field such as psychology is complicated because successful outcomes are based on statistics. A successful experiment in psychology is generally one that rejects the null hypothesis, but a key lesson from the TES analysis is that the rate of successful replication must reflect the power of the experiments. Having experiments with moderate or low power consistently reject the null hypothesis is cause for concern rather than celebration. For many of the articles in Table 2, reporting one or two unsuccessful but theoretically relevant experimental results would have blunted the TES analysis. Of course, even though reporting unsuccessful experimental outcomes may remove the appearance of bias, it may not strengthen the argument for the theoretical conclusion because the unsuccessful outcomes may contradict the theory.

Not every experiment is methodologically sound, and some experiments (even if methodologically sound) do not clarify the status of a theoretical idea. There is little reason to publish such experimental results, whether they are statistically significant or not. Unfortunately, in day-to-day scientific practice it is quite easy to interpret an unsuccessful outcome as being irrelevant to the theory or as being methodologically flawed and therefore not worth reporting. Such an attitude may reflect the conventional wisdom that non-significant results do not provide useful information because it is not possible to prove the null. Although there is truth to that conventional wisdom, reporting only significant outcomes misrepresents the magnitude of effects and can make even true null effects appear to be non-zero. In a variation of this approach, researchers may abort experiments that appear to not be working and instead focus resources elsewhere. A researcher who suppresses such an incomplete experimental result can honestly say that they do not know what would happen for a completed experiment; but if only seemingly successful experiments are run to completion, then the resulting findings are almost surely biased. A set of such experiments will tend to have an excess of success.

### Gathering More Data is Not Always Better

Statistical inference almost always improves with larger samples, which suggests that researchers using hypothesis testing will improve their conclusions by increasing sample sizes. Although unclear results should motivate scientists to gather more data and thereby reveal the truth, this idea does not work well in psychological science because the standard logic of frequentist hypothesis testing is valid only when the sample size is fixed prior to analysing data.

Despite this limitation in hypothesis testing, a common request by reviewers and editors is for authors to add more participants and see if a weak (say, *p* = 0.07) result might become significant. Reviewers almost never request that authors add more participants to see if data with a moderate (say, *p* = 0.03) result might become non-significant. Over multiple experiments, these requests, or similar “optional stopping” by authors, produce a bias that exaggerates effect sizes and replication rates of measured effects [44]–[46]. Gathering additional data can lead to misleading *p* values, because the Type I error rate increases rapidly with additional tests.

An experiment that stops as soon as it finds a significant result tends to produce *p* values that are just below the significance criterion. Indeed, for many of the studies in Table 2, relevant significant outcomes have *p* values just below 0.05 [43], which tends to produce a set of experiments with excess success.

### The Data Should Not Always Define the Theory

A principle tenet of science is that a theory must change (or be rejected) to reflect new data. The principle is true, but if the precision of empirical measurements is poor, then theories defined by the data statistics largely reflect noise.

**Figure 1:**
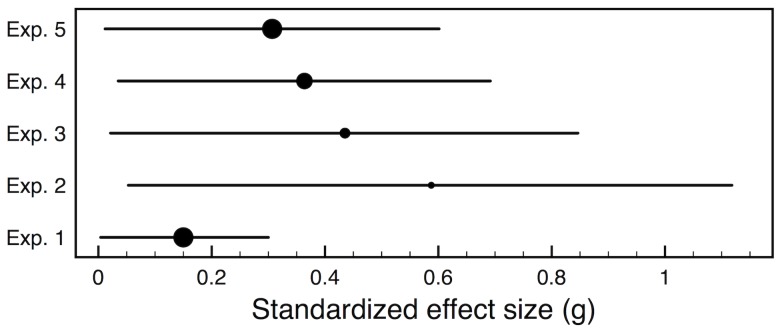
The circles mark the standardized effect size for the key findings in five experiments [40]. Each horizontal line indicates the range of a 95% confidence interval for the effect size. The diameter of a circle indicates the relative sample size of the experiment, with the largest sample size being 179.

Consider the precision of the measured effects reported by Gervais and Norenzayan [40], which is representative of many of the articles in Table 2. Figure 1 characterizes the 95% confidence interval [47] for each experiment's standardized effect size (Hedges' *g*). The confidence intervals around these effect size estimates stretch from almost zero to above 1.2. The breadth of these confidence intervals indicates that most of the experiments give little clarity about the true size of the measured effects. A theory that perfectly matches the mean data may be the best fit by conventional statistical criteria (e.g., maximum likelihood); but if the data are noisy then a best-fitting theory is not necessarily a good-fitting theory [48], [49]. When imprecise experimental results are pooled together through meta-analysis or as converging evidence, they can constrain theoretical ideas in important ways, but the validity of such pooling requires the experiment set to be unbiased.

Confusion about the relationship between data and theory is also reflected in what Kerr [50] called hypothesizing after the results are known (HARKing). Some researchers are so fixated on the 0.05 criterion that they take any significant result as something that should be included in a theory but judge any non-significant finding as irrelevant to the theory or as identification of a boundary condition (and thus part of the theory). Sometimes a theory that emerged from a dataset is presented as if it predicted the dataset, which is clearly a misrepresentation of the scientific process [51].

If many *p* values are close to the significance criterion, then HARKing will produce theoretical claims with a high risk of being based on noise. A TES analysis of such findings will often reveal excess success, reflecting the fact that an exact replication would be unlikely to produce the same pattern of significant and non-significant outcomes.

### Not All Confirmed Predictions Support a Theory

Perhaps no outcome of science is more convincing than when a theory makes a novel prediction that is verified by a new experiment. A common phrase in the articles contributing to Table 2 goes something like, “as predicted by the theory,” which is then followed by the report of a successful hypothesis test. Such verification of a prediction seems like strong validation of the theoretical ideas, but the belief in this validation is sometimes unjustified. The outcome of a statistical hypothesis test varies across random samples, which implies that the best any theoretical predictor can do is to estimate the probability of an experimental outcome. If the predicted outcome is to reject the null, this probability is power; and its estimation requires knowing an effect size, sample size, and analysis design. None of the articles in Table 2 described a theoretically motivated effect size, which means that the theories in those articles do not actually predict the outcome of a hypothesis test (even probabilistically). Thus, the consistently reported validation of theory predictions is actually a cause for concern.

What may have happened for some of the articles listed in Table 2 is that the authors had valid reasons to search for the existence of certain effects, but they did not convert such ideas into quantitative predictions about the outcome of a hypothesis test. Without a quantitative prediction, it is difficult to design an experiment that can convincingly demonstrate a prediction failure [52]. Given pressure to reduce costs, such experiments tend to be underpowered and thereby produce outcomes that are difficult for researchers to interpret. In such situations, experiments that happen to qualitatively match a predicted outcome may be given undue credence, since the observed outcome is partially due to chance, even while experiments that do not match a prediction are dismissed as being underpowered. With inadequate tests of a prediction, a scientist may strongly feel that he or she is following good scientific practice even while producing dubious support for their theory. Mistakes in data collection, statistical analysis, or reporting of relevant findings further magnify the discrepancy between a researcher's belief in the validity of a theoretical interpretation of the reported findings and the biased properties of those findings.

## Conclusions

Eighty-three percent of the Psychology/Education articles with four or more experiments that were published in *Science* (2005–2012) have an excess of success, which suggests that their results are too good to be true. Since scientists should be skeptical about those articles' theoretical claims, this high rate of bias is disturbing. It is unlikely that psychology uniquely faces these problems, as analyses suggest that at least some findings in neuroscience and medicine have similar problems [53]–[55], and these concerns may also apply to other fields that use statistics [56].

We would like to emphasize that the appearance of excess success does not establish intentional misconduct by the authors of an article. Given the problems identified above and in other discussions about statistics and publication practices in psychology [6], [7], [10], [46], [50], [51], [57]–[59], we believe that the appearance of excess success is often an honest mistake by authors who did not appreciate the inherent variability that should appear in their hypothesis test results. Such misunderstandings may lead to misinterpretations (e.g., concluding that a non-significant outcome is irrelevant and need not be reported) or over-interpretations (e.g., deriving a theory to match all reported outcomes) of experimental findings; and these errors lead to poor theories and excess success.

In terms of reform, we see promise in an approach that advises exploratory empirical work to focus on principles of estimation (e.g., [60]–[62]) and in a complementary approach that advises formal methods for model development and theory testing [59], [63], [64]. Discussions about empirical findings and theories in the published literature are an integral part of the scientific process, so we also see benefit to systems such as PubMed Commons and Pub Peer that encourage such discussions and to systems such as the Open Science Framework [65] that improve access to empirical data.

Overall, we believe that many of the current problems in psychology reflect misunderstandings about how to draw theoretical conclusions from statistical data. Moreover, we believe that these problems can be fixed and that psychological scientists will be receptive to the solutions.

## Supporting Information

Information S1 **TES analyses for individual articles.** This document provides a full analysis of each article listed in Table 2. It also explains why some articles could not be analyzed.
(PDF)

Information S2 **TES analysis calculations.** This compressed file contains a directory for every article in Table 2. Each directory includes a text file describing the location of the statistics taken from the article that were used for the TES analysis. It also includes a spreadsheet that summarizes the statistics, computes effect sizes (where appropriate), and lists the estimated success probability for each experiment. The directory also includes any *R* source code that was used to estimate success probability.
(ZIP)
