# Supplementary material for: Excess Success for Psychology Articles in the Journal Science
Source: PLoS One. 2014 Dec 4;9(12):e114255. doi: 10.1371/journal.pone.0114255 (PMC4256411; doi:10.1371/journal.pone.0114255)
Supplement: Information S1 — TES analyses for individual articles. This document provides a full analysis of each article listed in Table 2. It also explains why some articles could not be analyzed. (PDF) [file pone.0114255.s001.pdf]

Supplementary material for: Excess success for psychology articles in the journal *Science*

Authors: Gregory Francis*[1], Jay Tanzman[2], William J. Matthews[3]

Affiliations:

[1]Department of Psychological Sciences, Purdue University, USA and Brain Mind Institute, École Polytechnique Fédérale de Lausanne, Switzerland

[2] Independent statistician

[3]Department of Psychology, University of Cambridge, UK

*Correspondence to: gfrancis@purdue.edu

1. Estimating the Probability of Experimental Success

Even if an effect exists in a population, random samples drawn from that population will not always demonstrate evidence for the effect by satisfying statistical significance. Power is the probability of rejecting the null hypothesis for a given experimental design, sample size, and effect size. In some situations, support for a theoretical position is based on observed non-significant findings; and the probability of such an outcome can be computed as the complement of power.

Some power calculations are easily computed with software packages (Champely, 2009; Lenth, 2009; Faul et al., 2007), but when multiple tests are part of the original analysis it is sometimes only possible to calculate an upper limit of power. For many experimental results, the upper limit of the joint power across multiple tests is estimated with the lowest power across the tests. Thus, if three tests from one data set have powers of 0.8, 0.7, and 0.5, the smallest upper limit of the joint power is 0.5. This estimate likely overestimates the true power, since the conditional probability of the other two tests rejecting the null is unlikely to be the value one. If an article provides the correlations between measures from a single data set, it is possible to estimate the probability of multiple tests rejecting the null.

Some analyses place multiple restrictions on a single data set (e.g., a pattern of significant and non-significant main effects or interactions). The probability of selecting data that generate such patterns can sometimes be estimated with simulation experiments that repeatedly sample from populations with means and variances matching those reported in the published study. Such estimations are more common for between-subject designs than for within-subject designs, because statistical reports of the latter usually do not report the correlations between paired measurements. Simulated probabilities were always based on 100,000 simulated experiments that were then analyzed with the tests used in the original article. The proportion of simulated outcomes that reproduced the observed pattern

of significant and/or non-significant outcomes was an estimate of the probability of experimental success.

When estimating outcome success probabilities, the TES analyses always gave a benefit of the doubt to the original research. We assumed that the original hypothesis tests were appropriate for the reported data (e.g., the data were randomly sampled from normal distributions with a common variance) and were meaningfully related to the derived theoretical conclusions. When the original report did not specify the sample sizes for different groups, the TES analysis assumed a nearly balanced design, which tends to maximize power.

One surprisingly difficult aspect for some analyses was how to identify the criterion for indicating statistical significance. The norm is to use $P<.05$, but a recalculation of a P value sometimes indicates that authors rounded down values of $P=.055$ (or higher). Such rounding down misrepresents the data, so it would be appropriate to be skeptical about the conclusions that were based on such statistical reports. On the other hand, the .05 criterion is arbitrary and a result with $P=.051$ is nearly as good/bad as a result with $P=.049$. Similarly, it is not uncommon for authors to report "marginal significance" when $P<.1$ and then use the result to support their theoretical claims. The criterion for significance is important for the TES analysis because a larger criterion for the P value makes it easier to reject the null hypothesis and thereby increases statistical power.

In the TES analyses described below, we tried to make a good faith interpretation of the authors' intentions regarding statistical significance. Unless stated otherwise, the $P<.05$ criterion was assumed. If statistical significance was claimed with what appeared to be rounding down of the P value (e.g., the text reports $P=.05$ but a recalculation gives $P>.05$), then the criterion for significance was judged to be just above the observed P value. If marginal significance was claimed and the result was used to support the theoretical claims, then the criterion was judged to be $P<.1$. In the tables below that describe the TES analyses, the reported effect size is Hedges' *g*, (Hedges, 1981) unless specified otherwise.

2. Selection of Articles for the TES Analysis

From the *Science* subject collection on-line, we downloaded all "original research" articles and their supplementary material that were classified as "Psychology" or "Education" for years 2005-2012. A total of 133 articles met these criteria. Each article and its supplemental material were then checked to determine if they contained four or more studies. There were 25 such articles classified as Psychology and one such article classified as Education.

Each of the 26 articles with four or more studies was examined to see if the TES analysis could be performed with the information provided in the article and supplemental material. Articles were excluded from the TES analysis if it was not possible to compute success probabilities for at least four studies. Table S1 lists the eight excluded articles and the reasons for their exclusion from the TES analysis. Further details are available upon request.

Table S1: These articles included four or more studies but could not be fully analysed because it was not possible to estimate success probabilities for at least four studies.

Year	Authors	Reason for not being fully analysed by TES
2012	Aviezer, Trope & Todorov	Several ANOVAs are within subject designs that do not report effect sizes or correlations between measures. Success probabilities cannot be estimated for these ANOVAs.
2012	Duncan, Sadanand & Davachi	The results of Experiment 1a were used in the analyses of Experiments 1b and 2, which prohibits estimating success probabilities for at least one of the experiments. This leaves only three success probability estimates.
2012	Koriat	Analyses included standardization of data sets prior to dyad pairing. Estimating success probabilities would require access to the raw data and analysis scripts.
2011	Sparrow, Liu & Wegner	The statistical analyses contain errors that prohibit computing success probabilities. The *F* value in Experiment 1 does not match the reported means and standard deviations. The reported P value in Experiment 2 does not agree with the reported *F* value. The standard deviations reported for Experiment 4 do not match the standard errors in Figure 2.
2011	Thomsen, Frankenhuis, Ingold-Smith & Carey	Hypothesis tests are both within experiments and between experiments (e.g., Exp. 1 vs. 3 and Exp. 2 vs. Exps. 4 and 5). Given the within-subject designs, it is not possible to estimate success probabilities both within and across experiments.
2009	Chapman, Kim, Susskind & Anderson	Experiment 1b does not use a hypothesis test, so there is no success probability estimate. This leaves only three experiments with success probabilities.
2008	Maya-Ventencourt, Sale, Viegi, Baroncelli, De Pasquale, O'Leary, Castrén & Maffei	Hypothesis tests report only vague P values rather than test statistics. Cannot estimate success probabilities.
2006	Mulcahy & Call	The data in Experiment 2 do not match a reported P value. Experiments 1, 3, and 4 reported multiple tests within each experiment and across experiments. Because variables in these tests are correlated, it is not possible to estimate success probabilities for the different tests, so there end up being only three success probabilities estimates.

The following sections provide details of the TES analysis for each article in Table 2 of the main article. This supplemental material also includes text files that identify the tests taken from each article, spreadsheets that calculate effect sizes, and R scripts (R Development Core Team, 2013) used to estimate success probabilities for complicated situations.

3. Dijksterhuis, Bos, Nordgren & van Baaren (2006) "On making the right choice: The deliberation-without-attention effect"

Dijksterhuis *et al.* (2006) reported four experiments purporting to show that people make better decisions for simple circumstances with conscious thinking, but that unconscious thinking promotes better decisions for complex circumstances. Table S2 summarizes the statistics that contributed to the TES analysis.

Experiment 1 compared the proportion of subjects who made choices in one of four conditions. The proportions were extracted from Figure 1 in Dijksterhuis *et al.* (2006) and the sample sizes were estimated to produce these proportions. According to the conclusions in Dijksterhuis *et al.* (2006), a successful outcome in Experiment 1 required a significant interaction, one significant comparison (conscious thinkers in simple vs. complex conditions), and one non-significant comparison (unconscious thinkers in simple vs. complex conditions). The probability of the experiment to produce this pattern was estimated with simulated experiments.

Experiment 2 had a design similar to Experiment 1, but only the interaction statistic was reported. It is not clear if the differences across tasks were statistically significant, but Dijksterhuis *et al.* (2006) treated such effects as unrelated to the success of the experimental result, so they were not used to estimate success probability. The test statistic in Table S2 is an *F* value.

Table S2. Statistical properties of the Dijksterhuis *et al.* (2006) experimental findings.

	n	Test Statistic	p	Effect Size	Probability of Success
Exp. 1	20, 19 21, 20	Multiple tests	--	--	.356
Exp. 2	24, 25	5.63	.022	0.667	.628
Exp. 3	49	2.13	.038	0.300	.538
Exp. 4a	13, 14	6.52	.017	0.954	.663
Exp. 4b	13, 14	6.12	.021	0.924	.635
P_{TES}					.051

Experiment 3 was based on a regression showing that the interaction of complexity and amount of thought predicted post-choice satisfaction. The test statistic in Table S2 is a *t* value. Experiment 4 had hypothesis tests for two different customer types, who showed opposite behavior patterns. Since the two groups were independent samples, they were

treated as Experiments 4a and 4b, with separate success probability calculations. The test statistics in Table S2 are *F* values.

Dijksterhuis *et al.* (2006) reported that each of the studies produced a pattern of results that supported their theoretical stance. If the theory is correct, and the population effects are as estimated by the samples, then the probability of getting five studies like these producing the desired pattern is the product of the success probabilities: $P_{TES} = .051$. Since this value is less than the .1 criterion, readers should be skeptical about the reported experimental results as they relate to the theory.

4. Analysis of Vohs, Mead & Goode (2006) "The Psychological Consequences of Money"

Vohs *et al.* (2006) reported nine experiments purporting to show that money induces a self-sufficient orientation. Table S3 summarizes the statistics that contributed to the TES analysis.

Experiment 1 compared participants primed with money against participants primed with play money and participants in a control condition. The statistical analysis included a significant omnibus ANOVA and significant contrasts between the control and each of the other conditions. There was a predicted non-significant difference between the money and play money conditions. The probability of the data generating this pattern of results was estimated with simulated experiments.

Experiment 2 assigned participants to a high money and a low money condition. The dependent variable was the length of time working on a problem before asking for help. As predicted, participants in the high money condition worked longer. The success probability of the experiment is quite low because the statistical result just barely satisfied the conditions for statistical significance (P=0.05). Experiments 3, 4, and 6 had similar designs with different priming conditions and dependent variables. The statistics in Table S3 are *t* values.

Experiment 5 measured helpfulness by counting how many spilled pencils a participant picked up. Hypothesis tests showed a significant omnibus ANOVA across three conditions. Contrasts showed significant differences between a money condition and each of two control conditions. Success probability was estimated with simulated experiments. Experiment 7 had a similar design, but the analysis did not include the main effect from the ANOVA. Experiment 8 also had a similar design, and included the main effect for the ANOVA among its other tests.

Experiment 9 also had a similar design but used χ^2 tests to compare the proportion of participants willing to work on a task with a co-worker. Success probability was estimated with simulated experiments.

Vohs *et al.* (2006) reported that each of the studies produced the pattern of results that support their theoretical stance. If the theory is correct, and the population effects are as estimated by the samples, then the probability of getting studies like these to all produce the desired pattern is the product of the success probabilities: $P_{TES} = .002$. Since this value is less than the .1 criterion, readers should be skeptical about the reported experimental results as they relate to the theory.

Table S3: Statistical properties of the Vohs *et al.* (2006) experiments.

	n	Test Statistic	p	Effect Size	Probability of Success
Exp. 1	17, 17, 18	Multiple tests	--	--	.476
Exp. 2	19, 19	2.03	.050	0.645	.490
Exp. 3	20, 19	2.06	.046	0.646	.502
Exp. 4	22, 22	2.13	.039	0.631	.533
Exp. 5	11, 11, 11	Multiple tests	--	--	.403
Exp. 6	22, 22	2.13	.039	0.631	.533
Exp. 7	12, 12, 12	Multiple tests	--	--	.458
Exp. 8	21, 20, 20	Multiple tests	--	--	.453
Exp. 9	12, 13, 12	Multiple tests	--	--	.729
P_{TES}					.002

5. Analysis of Zhong & Lijenquist (2006) "Washing Away Your Sins: Threatened Morality and Physical Cleansing"

Zhong & Lijenquist (2006) reported four experiments purporting to show that a threat to morality induced a desire for physical cleansing. Table S4 summarizes the statistics that contributed to the TES analysis. A slightly different version of the TES analysis was provided in Francis (2012d), which pooled similar effects and also considered experimental data from two failed replication experiments (Fayard, Bassi, Bernstein & Roberts, 2009). The conclusions are similar across these different analyses.

In Experiment 1 participants recalled either an unethical deed or an ethical deed from their past. They then completed a word fragment task with items that could be finished as cleansing-related or unrelated words. Participants generated more cleansing-related words if they had recalled an unethical deed. Table S4 gives the *F* statistic for the hypothesis test.

In Experiment 2 participants copied a story that described an ethical or unethical character. They then rated the desirability of cleansing-related or unrelated products. Participants in the unethical condition gave higher ratings to the cleansing products. Table S4 gives the *F* statistic.

Experiment 3a used the initial task from Experiment 1 and then offered a choice of a gift. Participants in the unethical condition were more likely to select a cleansing product. The test statistic listed in Table S4 is a χ^2 value and the effect size is Cohen's *h*. The supplemental material described Experiment 3b, where participants indicated a preference between the gifts. The desired result was a non-significant difference from 0.5. The success probability in Table S4 indicates the probability of not rejecting the null hypothesis.

Table S4: Statistical properties of the Zhong & Lijenquist (2006) experiments.

	n	Test Statistic	p	Effect Size	Probability of Success
Exp. 1	30, 30	4.26	.044	0.526	.517
Exp. 2	13, 14	6.99	.014	0.987	.693
Exp. 3a	16, 16	4.57	.030	0.776	.593
Exp. 3b	15	0.07	.796	--	.959
Exp. 4a	22, 23	2.94	.047	0.502	.505
Exp. 4b	22, 23	0.25	.310	0.146	.923
P_{TES}					.095

In Experiment 4a participants completed the unethical condition from Experiment 1 and then either washed their hands or not. Participants who washed their hands reported reduced moral emotions compared to participants who did not wash their hands. The test statistic in Table S4 is an *F* value and the P value reflects a one-tailed test; these conditions were part of the success probability estimate (a one-tailed test is more powerful than a two-tailed test). This experiment also reported a significant effect of washing condition on volunteerism. The joint success probability of both findings must be less than the success probability of either finding alone, so the success probability estimate given in Table S4 likely overestimates the true success probability. Another hypothesis test (Experiment 4b) found that hand washing did not influence nonmoral emotions. Zhong & Lijenquist (2006) treated this test as an independent analysis, and this seems appropriate given the theoretical interpretation of the data. Thus it is appropriate to consider the probability of a random sample producing a non-significant result, and Table S4 provides this estimate.

Zhong & Lijenquist (2006) reported that each of the studies produced a pattern of results that support their theoretical stance. If the theory is correct, and the population effects are as estimated by the samples, then the probability of getting studies like these to all produce the desired pattern is the product of the success probabilities: $P_{TES} = .095$. Since this value is less than the .1 criterion, readers should be skeptical about the reported experimental results as they relate to the theory.

6. Analysis of Wood, Glynn, Phillips & Hauser (2007) "The Perception of Rational, Goal-Directed Action in Nonhuman Primates"

Wood *et al.* (2007) reported two experiments, each with three independent sets of primates, that purported to show that primates could distinguish between goal-directed and accidental actions by another individual. Table S5 summarizes the statistics that contributed to the TES analysis.

In Experiment 1, a human experimenter interacted with two containers in either a goal-directed or an accidental manner. The primate's task was to subsequently inspect the containers, and the dependent measure was the amount of time spent with the container that received the goal-directed interaction from the experimenter. Statistical results were reported for three different species. Different one-tailed tests were used for each species

and the success probability calculations take these test properties into account. Tamarins were tested with an *F* test. Rhesus monkeys were tested with a χ^2 test, and the effect size in Table S5 is Cohen's *h*. Chimpanzees were tested with a Wilcoxon signed ranks test.

Experiment 2 was similar in design and analysis, but used a different interaction to indicate goal-directed and incidental behavior by the experimenter. The tamarin version of the experiment was between-subjects. A non-standard criterion of P=0.06 was used by Wood *et al.* (2007) to conclude statistical significance, so the same value was used for the success probability analysis.

Wood *et al.* (2007) reported that each of the studies produced the pattern of results that support their theoretical stance. If the theory is correct, and the population effects are as estimated by the samples, then the probability of getting studies like these to all produce the desired pattern is the product of the success probabilities: P_{TES} = .051. Since this value is less than the .1 criterion, readers should be skeptical about the reported experimental results as they relate to the theory.

Table S5: Statistical properties of the Wood *et al.* (2007) experiments.

	n	Test Statistic	p	Effect Size	Probability of Success
Exp. 1, tamarins	10	3.57	.046	0.546	.481
Exp. 1, rhesus	20, 20	4.29	.019	0.675	.688
Exp. 1, chimpanzees	25	-2.02	.021	-0.404	.607
Exp. 2, tamarins	10, 15	2.60	.060	0.636	.485
Exp. 2, rhesus	32, 32	10.47	<.001	0.848	.960
Exp. 2, chimpanzees	25	-1.87	.031	-0.347	.551
P_{TES}					.051
Replication Exp. 1	26	0.692	.038	--	.597
P_{TES} including replication					.031

Given concerns about some of the senior (last) author's other publications, which were eventually concluded to contain fraudulent data, Wood and Hauser (2011) described a supplemental experiment that replicated Experiment 1, and the reported replication was widely considered to support the findings in Wood *et al.* In fact, the successful replication does the opposite because it is even less believable that all experiments would reject the null with such modest success probabilities. The replication experiment reported that 18 out of 26 rhesus monkeys selectively inspected the targeted container. A binomial test gives P=0.04, but the estimated success probability of such a test is only 0.597. The probability of the original and replication experiments all rejecting the null is P_{TES} = .031.

7. Analysis of Whitson & Galinsky (2008) "Lacking Control Increases Illusory Pattern Perception"

Whitson and Galinsky (2008) reported six experiments purporting to show that lack of control increased perception of illusory patterns. Table S6 summarizes the statistics that contributed to the TES analysis.

In Experiment 1, participants were assigned to control or lack-of-control conditions and need for structure was measured with a standard scale. Participants without control reported increased need for structure. The test statistic in Table S6 is a *t* value. Experiment 2 used the same conditions but asked participants to report whether they saw any traces of an image in a noisy pattern. For noise patterns that did not have an embedded picture, participants in the lack-of-control condition were more likely to report seeing a pattern. The test statistic in Table S6 is a *t* value. Support for the theoretical conclusion was determined with a marginally significant P value.

Experiment 3 varied the method of inducing control and then had participants judge relations between events in various scenarios. Participants in the lack-of-control condition reported strong connections between events. The test statistic in Table S6 is a *t* value. This experiment also reported a significant difference in measured worry. Since the data for the two measures come from the same set of participants, the scores should be correlated, which makes it impossible to estimate the success probability of both test results. The success probability given in Table S6 provides an upper bound for the joint success probability of both effects.

Experiment 4 was similar to Experiment 2, but used a different inducement of control. The test statistic in Table S6 is a *t* value. This study also reported a significant difference for another measure (likelihood of conspiracy), so the success probability in Table S6 establishes an upper limit estimate on the joint success probability.

Experiment 5 manipulated control by describing a stock market as being volatile or stable. Participants indicated whether they would invest in a described company. An illusory perception about the quality of the company was introduced by varying the number of positive and negative statements about each company. More negative statements were predicted to carry more weight in a volatile market than a stable marked. As predicted, fewer participants in a volatile market invested in the company with more negative statements compared to participants in a stable market. The test statistic in Table S6 is a χ^2 value and the effect size is Cohen's *h*. This experiment also reported several other tests that agreed with the theoretical conclusions, so the given success probability provides an upper limit on the joint success probability.

Experiment 6 explored whether self-affirmation would reduce illusory pattern perception by assigning participants to three conditions: lack-of-control without self-affirmation, lack-of-control with self-affirmation, and baseline. There were several dependent variables, a key one being ratings about the likelihood of a conspiracy. The test statistic in Table S6 reports a *t* value for a contrast between the lack-of-control without self-affirmation condition against the other two conditions. Since tests for other dependent variables were also significant, the success probability given in Table S6 provides an upper limit for the joint success probability.

Table S6: Statistical properties of the Whitson and Galinsky (2008) experiments.

	n	Test Statistic	p	Effect Size	Probability of Success
Exp. 1	14, 15	2.11	.044	0.762	.507
Exp. 2	18, 18	1.76	.087	0.574	.517
Exp. 3	21, 20	2.03	.049	0.622	.493
Exp. 4	12, 13	2.18	.040	0.844	.524
Exp. 5	20, 24	4.94	.026	-0.691	.626
Exp. 6	17, 17, 16	2.08	.043	0.579	.517
Footnote 35	22, 23, 21, 20	Multiple tests	--	--	.357
P_{TES}					.008

An additional experiment was described in Footnote 35 that explored how conspiracy perception was related to control in a two by two design that varied control (present, absent) and focus (self, other). The outcomes relevant to the theoretical ideas included a main effect of control, no interaction, and significant effects of control for both self and other conditions. The joint success probability of these tests was estimated with simulated experiments. There is a discrepancy between the degrees of freedom given for the tests and the sample size listed in the supplemental material of Whitson and Galinsky (2008). The TES analysis was based on sample sizes derived from the degrees of freedom.

Whitson and Galinsky (2008) reported that each of the studies produced the pattern of results that support their theoretical stance. If the theory is correct, and the population effects are as estimated by the samples, then the probability of getting studies like these to all produce the desired pattern is the product of the success probabilities: $P_{TES} = .008$. Since this value is less than the .1 criterion, readers should be skeptical about the reported experimental results as they relate to the theory.

8. Analysis of Mehta & Zhu (2009) "Blue or Red? Exploring the Effect of Color on Cognitive Task Performances"

Mehta and Zhu (2009) reported eight experiments purporting to show that the color red induces avoidance and detail while blue induces approach and creativity. Table S7 summarizes the statistics that contributed to the TES analysis. Every experiment contained multiple between-subjects conditions and included several hypothesis tests. For all experiments, success probability was estimated with simulated experiments. Some experiments included the correlation between two dependent variables, and this information was included in the success probability estimation simulations.

In Experiment 1, participants were assigned to a blue, red, or neutral background color condition while reporting brand preferences among choices that highlighted avoiding a negative outcome or highlighted approaching a positive outcome. *t* tests reported significant differences between the blue and red conditions and between the blue and neutral conditions. There were additional tests related to a second task (anagram problems)

that were completed by the same participants, so the success probability listed in Table S7 provides an upper limit on the joint success probability of both tasks.

Experiments 2a and 2b had a similar design and analysis and measured false recalls for a detailed-oriented memory task (Experiment 2a) or performance on a creativity tasks (Experiment 2b). Again, participants in the blue condition scored higher than those in the red or neutral conditions. Experiment 2b included some additional measures that were also consistent with the theoretical ideas, so the success probability in Table S7 is an upper limit on the joint success probability of the findings.

Experiments 3a and 3b also had a similar design that measured accuracy on a proofreading task (Experiment 3a) or creativity (Experiment 3b). Support for the theoretical ideas was based on a main effect and contrasts that indicated better performance for participants in the red condition for the proofreading task but better creativity for participants in the blue condition.

Experiment 4 had participants combine either red or blue parts to create a child's toy. Black-and-white versions of the resulting drawings were evaluated for novelty and practicality. Toys created with red parts were judged more practical while toys created with blue parts were judged more novel. Mehta and Zhu (2009) reported that the correlation between these judgments was r=0.29, so the success probability estimation simulation included both hypothesis tests. A successful outcome required both tests to produce significant results.

Table S7: Statistical properties of the Mehta and Zhu (2009) experiments.

	n	Test Statistic	p	Effect Size	Probability of Success
Exp. 1	23, 23, 23	Multiple tests	--	--	.493
Exp. 2a	35, 34, 34	Multiple tests	--	--	.475
Exp. 2b	35, 35, 35	Multiple tests	--	--	.526
Exp. 3a	18, 18, 18	Multiple tests	--	--	.436
Exp. 3b	22, 21, 21	Multiple tests	--	--	.524
Exp. 4	21, 21	Multiple tests	--	--	.223
Exp. 5	38, 38, 39, 39	Multiple tests	--	--	.353
P_{TES}					.002

Experiment 5 varied background color as participants evaluated a camera advertisement that emphasized either detailed-oriented processing or remotely related associations. Experimental success was for a significant interaction and two significant contrasts. Participants with the red background gave more favorable evaluations when the ad emphasized details, but the reverse was found for participants with a blue background. Statistical significance was concluded for the former case with P<.07, and the same criterion was used for the success probability estimation.

Experiment 6 investigated whether participants were aware of the reported effects of red and blue colors. The findings indicated that participants generally believed that a blue color would promote creativity (consistent with the other reported studies) but also

that a blue color would promote detailed processing (inconsistent with the other reported studies). It is not clear what pattern of findings would invalidate the theoretical idea that participants are unaware of the true effect of colors (e.g., null findings would be consistent with the theory), so this study was not included in the TES analysis.

Mehta and Zhu (2009) reported that each of the studies produced the pattern of results that support their theoretical stance. If the theory is correct, and the population effects are as estimated by the samples, then the probability of getting studies like these to all produce the desired pattern is the product of the success probabilities: $P_{TES} = .002$. Since this value is less than the .1 criterion, readers should be skeptical about the reported experimental results as they relate to the theory.

9. Analysis of Paukner, Suomi, Visalberghi & Ferrari (2009) "Capuchin Monkeys Display Affiliation Toward Humans Who Imitate Them"

Paukner *et al.* (2009) reported six experiments purporting to show that imitation promotes affiliation in nonhuman primates. Table S8 summarizes the statistics that contributed to the TES analysis. Experiments 1-5 are based on one-sample *t* tests, while the final experiment (described in the supplementary material) uses a Wilcoxon signed ranks test.

Experiments 1, 2, and 4 varied how a human experimenter imitated a monkey and how affiliation was measured. They all showed results that just satisfied the criterion for statistical significance. Experiments 3 and 5 predicted (and found) null results. For these latter experiments the success probability value listed in Table S8 is the probability that a sample would not reject the null hypothesis. For all experiments there were several other tests that supported the theory, thus the success probabilities in Table S8 should be considered upper limits of the joint success probability of each experiment.

Experiment S1 was a check on whether proximity of a monkey to a human was a proper measure of affiliation. The reported finding (a significant one-tailed difference in time interacting with different animal care technicians) was important for validating the measure used in the other experiments.

Table S8: Statistical properties of the Paukner *et al.* (2009) experiments.

	**n**	**Test Statistic**	**p**	**Effect Size**	**Probability of Success**
Exp. 1	11	2.23	.050	0.621	.460
Exp. 2	10	2.29	.048	0.662	.464
Exp. 3	11	1.56	.150	0.434	.744
Exp. 4	10	2.30	.047	0.665	.467
Exp. 5	10	0.49	.636	0.142	.931
Exp. S1	7	-2.02	.022	-0.763	.536
P_{TES}					.037

Paukner *et al.* (2009) reported that each of the studies produced the pattern of results that support their theoretical stance. If the theory is correct, and the population

effects are as estimated by the samples, then the probability of getting studies like these to all produce the desired pattern is the product of the success probabilities: $P_{TES} = .037$. Since this value is less than the .1 criterion, readers should be skeptical about the reported experimental results as they relate to the theory.

10. Analysis of Weisbuch, Pauker & Ambady (2009) "The Subtle Transmission of Race Bias via Televised Nonverbal Behavior"

Weisbuch *et al.* (2009) reported seven experiments purporting to show that televised nonverbal behaviors influence bias among viewers. Table S9 summarizes the statistics that contributed to the TES analysis.

In Experiment 1, participants provided a rating of how much a character in a video clip liked another unseen character in the video scene. All videos were derived from television shows. The *F* statistic in Table S9 tests the difference in likeability ratings between videos where the unseen character was black or white.

Experiment 2 reported a predicted correlation between exposure to the nonverbal biases and scores on an implicit association test (IAT). The effect size in Table S9 is a Pearson's correlation. Several other correlations also supported the theoretical claims, so the success probability estimate in Table S9 provides an upper limit of the joint success probability.

Experiments 3a and 3b controlled for some possible confounds and reported significant differences in IAT scores for participants viewing clips that presented nonverbal cues with either pro-black or pro-white characters. The test statistics are *F* values, which were based on differences across matched videos (same character in a positive or negative setting) rather than on differences across participants in different conditions. For Experiment 3a, the reported P=0.05 value is rounded down from 0.053. Success probability was estimated by supposing that statistical significance was based on a criterion of 0.053.

Table S9: Statistical properties of the Weisbuch *et al.* (2009) experiments.

	n	Test Statistic	p	Effect Size	Probability of Success
Exp. 1	23	4.30	.050	0.417	.482
Exp. 2	53	--	.047	0.280	.529
Exp. 3a	60	3.91	.053	0.256	.495
Exp. 3b	32	4.75	.037	0.376	.540
Exp. 4	19, 19, 18	Multiple tests	--	--	.395
P_{TES}					.027

Experiment 4 measured reports of racial prejudice for participants exposed to pro-black nonverbal bias, pro-white nonverbal bias, or a control condition. The analysis involved a significant main effect of condition, and significant contrasts between the pro-white and pro-black conditions and between the pro-black and control conditions. This

experiment included many other measures and successful hypothesis tests, but given the within-subject nature of the measures, it is not possible to estimate the joint success probability of the multiple outcomes. The success probability estimate in Table S9 provides an upper limit on the joint success probability.

Weisbuch *et al.* (2009) reported that each of the studies produced the pattern of results that support their theoretical stance. If the theory is correct, and the population effects are as estimated by the samples, then the probability of getting studies like these to all produce the desired pattern is the product of the success probabilities: $P_{TES} = .027$. Since this value is less than the .1 criterion, readers should be skeptical about the reported experimental results as they relate to the theory.

11. Analysis of Ackerman, Nocera & Bargh (2010) "Incidental Haptic Sensations Influence Social Judgments and Decisions"

Ackerman *et al.* (2010) reported six experiments purporting to show that physical touch experiences influence impressions and decisions about seemingly unrelated situations. Table S10 summarizes the statistics that contributed to the TES analysis.

Experiment 1 reported two measures of impression formation that were modestly correlated with each other (r=.36; from supplemental material). Each measure was tested for differences between participants carrying heavy or light clipboards. The joint success probability of both effects was estimated with simulated experiments.

Experiment 2 showed a similar effect of clipboard type for judgments of several social issues. The test statistic in Table S10 is an *F* value. Experiments 3 and 4 investigated the effect of interacting with rough or smooth puzzle pieces on subsequent judgments about social interactions. The reported test statistics are *F* values.

Experiments 5 and 6 investigated how haptic experiences with hardness influenced judgments about stability and rigidity. The test statistics in Table S10 are *F* values. Experiment 6 included several other significant effects for related judgments, so the estimated success probability value should be interpreted as an upper limit.

Table S10: Statistical properties of the Ackerman *et al.* (2010) experiments.

	n	Test Statistic	p	Effect Size	Probability of Success
Exp. 1	26, 28	Multiple tests	--	--	.323
Exp. 2	21, 22	5.46	.024	0.700	.610
Exp. 3	33, 31	5.15	.027	0.561	.598
Exp. 4	21, 21	4.45	.041	0.639	.524
Exp. 5	25, 24	4.52	.039	0.598	.536
Exp. 6	34, 34	4.30	.042	0.497	.524
P_{TES}					.017

Ackerman *et al.* (2010) reported that each of the studies produced the pattern of results that support their theoretical stance. If the theory is correct, and the population

effects are as estimated by the samples, then the probability of getting studies like these to all produce the desired pattern is the product of the success probabilities: $P_{TES} = .017$. Since this value is less than the .1 criterion, readers should be skeptical about the reported experimental results as they relate to the theory.

12. Analysis of Bahrami, Olsen, Latham, Roepstorff, Rees & Frith (2010) "Optimally Interacting Minds"

Bahrami *et al.* (2010) reported four experiments purporting to show that a pair of communicating participants had better visual acuity than either participant alone. Table S11 summarizes the statistics that contributed to the TES analysis.

Each experiment compared the sensitivity (slope of a psychometric function) of a dyad of participants against the more sensitive participant's sensitivity or against a model-predicted sensitivity value. The test statistics in Table S11 are *t* values. The reported *n* refers to the number of dyads. For some experiments there were other tests, but the within subjects design of the experiments makes it impossible to estimate the joint success probability. In general, the reported effects are strong enough that the success probabilities in Table S11 are likely to be close to the joint success probability.

Bahrami *et al.* (2010) reported that each of the studies produced the pattern of results that support their theoretical stance. If the theory is correct, and the population effects are as estimated by the samples, then the probability of getting studies like these to all produce the desired pattern is the product of the success probabilities: $P_{TES} = .332$. Since this value is larger than the .1 criterion, the TES analysis does not indicate that readers should be skeptical about the reported experimental results as they relate to the theory.

Table S11: Statistical properties of the Bahrami *et al.* (2010) experiments.

	**n**	**Test Statistic**	**p**	**Effect Size**	**Probability of Success**
Exp. 1	15	5.24	<.001	1.279	.996
Exp. 2	11	2.50	.031	0.696	.549
Exp. 3	14	5.91	<.001	1.487	.999
Exp. 4	11	2.68	.023	0.746	.608
P_{TES}					.332

13. Analysis of Kovács, Téglás & Endress (2010) "The Social Sense: Susceptibility to Others' Beliefs in Human Infants and Adults"

Kovács *et al.* (2010) reported eight experiments purporting to show that both adults and infants automatically encode the beliefs of other people. Table S12 summarizes the statistics that contributed to the TES analysis.

Each experiment involved presentation of movies that provided varied information about the location of a ball relative to an occluder. For some movies the participant and an agent in the movie had similar beliefs but in other movies the participant and agent had different beliefs about the ball location. Experiments 1, 2, 3, and a replication of

Experiment 1 described in the supplementary material measured detection latencies, while the other experiments measured looking time for infants. The test statistics in Table S12 for the former studies are *t* values, while the remaining test statistics are *F* values. Some experiments had additional tests, but the within subjects design prohibits calculation of joint success probability. Experiment 6 was predicted to produce a non-significant finding, and the success probability in Table S12 is the estimated probability of not rejecting the null hypothesis.

Kovács *et al.* (2010) reported that each of the studies produced the pattern of results that support their theoretical stance. If the theory is correct, and the population effects are as estimated by the samples, then the probability of getting studies like these to all produce the desired pattern is the product of the success probabilities: $P_{TES} = .021$. Since this value is smaller than the .1 criterion, readers should be skeptical about the reported experimental results as they relate to the theory.

Table S12: Statistical properties of the Kovács *et al.* (2010) experiments.

	**n**	**Test Statistic**	**p**	**Effect Size**	**Probability of Success**
Exp. 1	24	2.42	.024	0.478	.611
Exp. 2	24	2.10	.047	0.415	.494
Exp. 3	24	2.18	.040	0.430	.524
Exp. 1 replication	16	2.65	.018	0.629	.652
Exp. 4	14	5.65	.033	0.598	.554
Exp. 5	14	7.29	.018	0.679	.652
Exp. 6	14	0.05	.827	0.056	.946
Exp. 7	14	6.75	.022	0.654	.619
P_{TES}					.021

14. Analysis of Morewedge, Huh & Vosgerau (2010) "Thought for Food: Imagined Consumption Reduces Actual Consumption"

Morewedge *et al.* (2010) reported six experiments purporting to show that people ate less food if they had previously imagined eating the food. Table S13 summarizes the statistics that contributed to the TES analysis.

Experiment 1 used a between-subjects design that assigned participants to three different conditions that varied how many (0, 3, or 33) imagined actions involved eating M&M candies. The dependent variable was how many M&M candies were subsequently eaten from a bowl. The ANOVA analysis indicated a significant main effect, a significant contrast between the 33 and 0 conditions, a significant contrast between the 33 and 3 conditions, and a non-significant contrast between the 0 and 3 conditions. All of these results were deemed supportive of the theoretical idea. Success probability was estimated with simulated experiments. Table 1 in Morewedge *et al.* (2010) provides means and standard deviations, but the values do not match the reported *F* statistics for the hypothesis

tests. The success probability estimation simulations supposed that Table 1 correctly reports the mean values and then computed the pooled standard deviation value from the reported *F* values using algebra. The same approach was used for the other experiments, except for Experiments 4b and 5.

Experiment 2 dropped the 0 condition and introduced two changes. First, some participants imagined inserting quarters into a machine while other participants imagined eating M&Ms. Subgroups performed the imaginary tasks either 3 or 30 times. The analysis included a significant interaction, a significant contrast for the number of imaginings between the eating groups, and a predicted non-significant contrast for the number of imaginings between the quarters groups.

Experiment 3 had participants imagine eating or moving 3 or 30 M&Ms. The ANOVA reported a significant interaction, significant contrasts for eating 3 vs. 30 M&Ms and for eating vs. moving 30 M&Ms. The main effects were not significant.

Experiment 4 had participants imagine eating cheese or M&Ms. The dependent measure was the amount of subsequently eaten cheese. The analysis reported a significant interaction, a significant contrast between the 3 and 30 imagined cheese conditions, and a non-significant contrast between the 3 and 30 imagined M&M conditions.

Experiment 4b asked participants to predict the outcome of Experiment 4. A key result was that the participants incorrectly predicted that people who imagined eating 30 cubes of cheese would subsequently eat more cheese. The test statistic in Table S13 is a *t* value.

Experiment 5 used a within-subjects design to measure the difference in how much participants liked cheese before or after the imagination task. Table S13 reports the *F* value for one of several hypothesis tests. Because of the within-subjects design, it is not possible to estimate the joint success probability of all the tests, so the value given in Table S13 should be considered an estimate of the upper limit of the joint success probability.

Morewedge *et al.* (2010) reported that each of the studies produced the pattern of results that support their theoretical stance. If the theory is correct, and the population effects are as estimated by the samples, then the probability of getting studies like these to all produce the desired pattern is the product of the success probabilities: $P_{TES} = .012$. Since this value is smaller than the .1 criterion, readers should be skeptical about the reported experimental results as they relate to the theory.

Table S13: Statistical properties of the Morewedge *et al.* (2010) experiments.

	**n**	**Test Statistic**	**p**	**Effect Size**	**Probability of Success**
Exp. 1	17, 16, 16	Multiple tests	--	--	.449
Exp. 2	13, 13, 13, 12	Multiple tests	--	--	.332
Exp. 3	17, 17, 17, 17	Multiple tests	--	--	.434
Exp. 4	10, 10, 10, 11	Multiple tests	--	--	.380
Exp. 4b	80	3.09	.002	0.342	.856
Exp. 5	34, 34	4.82	.032	0.526	.571
P_{TES}					.012

15. Analysis of Halperin, Russell, Trzesniewski, Gross & Dweck (2011) "Promoting the Middle East Peace Process by Changing Beliefs About Group Malleability"

Halperin *et al.* (2011) reported four studies purporting to show that groups with a more malleable nature tended to compromise for peace. Table S14 summarizes the statistics that contributed to the TES analysis.

Each study measured or manipulated malleable belief, measured positive attitudes toward Palestinians or Israeli-Jews, and observed how those variables related to willingness to compromise. The main result for each study came from a moderation analysis and related statistics. Table S14 reports an *r* correlation value for Study 1 and *t* values for the other studies.

Halperin *et al.* (2011) reported that each of the studies produced the pattern of results that supports their theoretical stance. If the theory is correct, and the population effects are as estimated by the samples, then the probability of getting studies like these to all produce the desired pattern is the product of the success probabilities: $P_{TES} = .210$. Since this value is larger than the .1 criterion, the TES analysis does not indicate that readers should be skeptical about the reported experimental results as they relate to the theory.

Table S14: Statistical properties of the Halperin *et al.* (2011) experiments.

	n	Test Statistic	p	Effect Size	Probability of Success
Study 1	500	--	<.001	0.30	1.00
Study 2	38, 38	2.43	.020	0.552	.661
Study 3	30, 29	2.19	.033	0.563	.565
Study 4	26, 27	2.19	.033	.593	.562
P_{TES}					.210

16. Analysis of Ramirez & Beilock (2011) "Writing About Testing Worries Boosts Exam Performance in the Classroom"

Ramirez and Beilock (2011) reported four experiments purporting to show that writing down thoughts about an upcoming test improved performance on the test. Table S15 summarizes the statistics that contributed to the TES analysis.

Experiment 1 measured math accuracy for participants in a control or expressive writing condition. The analysis included several different *t* tests that reported no effect of condition for a pretest but a significant effect for a posttest. In addition, separate *t* tests reported that accuracy for controls dropped (they choked under pressure) from pretest to posttest, but rose for participants in the expressive writing condition. With the means and standard errors reported in Figure 1 of Ramirez and Beilock (2011) and with the *t* values reported in the text, it is possible to estimate the correlations between the pretest and posttest scores for each condition. Success probability of all of the reported effects was then estimated with simulated experiments.

Experiment 2 used a similar test, but had three conditions: control, expressive writing, and unrelated writing. The analysis reported five hypothesis tests. There was not a significant difference between groups on the pretest but there was a significant difference on the post-test. There was a significant drop pre to posttest for the control and unrelated groups, who did not show a significant difference with each other. The expressive writing group showed a significant increase between pretest and posttest, although the latter claim was based on rounding down P=0.054. This non-standard criterion was used as the definition of significance for estimating the success probability of this test. Figure 2 in Ramirez and Beilock (2011) provides the means and standard deviations of each condition, and these values can be combined with the reported t values to estimate the correlations between the pretest and posttest scores for each group. The success probability of all the hypothesis tests was then estimated with simulated experiments. The many constraints placed on the data are difficult to simultaneously satisfy, so the joint success probability is quite low.

Experiments 3 and 4 looked for evidence that the writing effects transferred to high-school students. Students were assigned to write about an upcoming exam or an unrelated topic. For each experiment individually and for their combined data, there was a significant correlation between test anxiety and exam scores for students writing on the unrelated topic. However, these correlations were non-significant for students writing about the exam. The correlations across conditions were significantly different. Since these tests are based on common datasets, the joint success probability was estimated with simulated experiments.

Ramirez and Beilock (2011) reported that each of the studies produced the pattern of results that support their theoretical stance. If the theory is correct, and the population effects are as estimated by the samples, then the probability of getting studies like these to all produce the desired pattern is the product of the success probabilities: $P_{TES} = .059$. Since this value is smaller than the .1 criterion, readers should be skeptical about the reported experimental results as they relate to the theory.

Table S15: Statistical properties of the Ramirez and Beilock (2011) experiments.

	**n**	**Test Statistic**	**p**	**Effect Size**	**Probability of Success**
Exp. 1	10, 10	Multiple tests	--	--	.539
Exp. 2	15, 16, 16	Multiple tests	--	--	.264
Exps. 3 and 4	26, 25, 30, 25	Multiple tests	--	--	.412
P_{TES}					.059

17. Analysis of Stapel & Lindenberg (2011) “Coping with Chaos: How Disordered Contexts Promote Stereotyping and Discrimination”

Stapel and Lindenberg (2011) reported six experiments purporting to show that disordered contexts promote stereotyping. Table S16 summarizes the statistics that contributed to the

TES analysis. An investigation into research fraud practiced by Stapel (Levelt, Noort & Drenth Committees, 2012) concluded that the data reported in this paper were fraudulent and the paper was retracted.

The first two studies were described as field experiments that measured stereotyping or discrimination in various ways for disordered or ordered situations. In Field experiment 1, discrimination was measured as the distance participants put between themselves and a black experimenter while completing a survey. The measure was taken during and after a sanitation strike. The *F* value in Table S16 compares the distances for the different environmental conditions. The study included several other significant tests as well, so the success probability value provides an upper limit.

Field experiment 2 reported that participants donated less money to a minority charity on a disordered street than on an ordered street. The test statistic in Table S16 is an *F* value. The study included several other significance tests, so the success probability is an upper limit.

Experiment 1, the first lab experiment, exposed participants to an ordered, neutral or disordered priming condition that presented photographs of events and scenes. The measurements were a need-for-structure scale and a stereotyping judgment. These measures were correlated with r=0.69. The analysis included a significant main effect for each measure. A covariance analysis found that the stereotyping measure was no longer significantly different once the need-for-structure measure was included as a covariate. All of these effects were included in the success probability estimate, which was based on simulated experiments. The standard errors in Figure 2 of Staple and Lindenberg (2011) did not match the reported *F* statistics. For the simulations, the standard deviation was derived from the means and the reported *F* values. Experiment 2 had a similar design but used a different method of priming. The analysis was the same as for Experiment 1, and success probability was estimated in the same way.

Experiment 3 induced order or disorder by having participants look at drawings of geometric shapes that were either arranged orderly or disorderly. Participants were also asked to perform a stereotyping task or a filler task. The dependent measure was need-for-structure. There was a significant interaction between the conditions, and the test statistic in Table S16 is an *F* value.

Table S16: Statistical properties of the Stapel and Lindenberg (2011) experiments.

	**n**	**Test Statistic**	**p**	**Effect Size**	**Probability of Success**
Field 1	40, 40	7.23	.009	0.595	.749
Field 2	24, 23	5.71	.021	0.686	.633
Exp. 1	16, 15, 16	Multiple tests	--	--	.526
Exp. 2	19, 19, 20	Multiple tests	--	--	.738
Exp. 3	14, 14, 15, 15	6.40	.014	0.655	.689
Exp. 1 replication	15, 15, 16, 15	Multiple tests	--	--	.594
P_{TES}					.075

The supplemental material in Stapel and Lindenberg (2011) reported a replication of Experiment 1, but also included a "cognitive load" condition. Success probability for this experiment was estimated in the same way as for Experiment 1.

Stapel and Lindenberg (2011) reported that each of the studies produced the pattern of results that support their theoretical stance. If the theory is correct, and the population effects are as estimated by the samples, then the probability of getting studies like these to all produce the desired pattern is the product of the success probabilities: $P_{TES} = .075$. Since this value is smaller than the .1 criterion, readers should be skeptical about the reported experimental results as they relate to the theory. This conclusion is redundant with the retracted status of the article, but it is valuable to recognize that the results warranted skepticism even without the fraud charges.

18. Analysis of Gervais & Norenzayan (2012) "Analytic Thinking Promotes Religious Disbelief"

Gervais & Norenzayan (2012) reported five experiments purporting to show that analytical thinking promoted religious disbelief. Table S17 summarizes the statistics that contributed to the TES analysis.

Experiment 1 reported three measures of religious belief, which were strongly correlated with each other. Statistically significant negative correlations with analytical thinking were reported for all three measures. Table S17 shows the weakest correlation value of the three measures, which establishes an upper limit on the success probability of producing all of the findings in Experiment 1.

Experiments 2-5 had similar designs with participants assigned to a control or analytical group and a measure of religiosity being the dependent variable. The test statistics in Table S17 are *t* values. An argument could be made that Experiments 2-5 all measure the same phenomena and that the effect sizes should be pooled. To stay consistent with the analyses considered for other articles, we do not report such a pooled analysis (but the main conclusion is unchanged).

Table S17: Statistical properties of the Gervais & Norenzayan (2012) experiments.

	n	Test Statistic	p	Effect Size	Probability of Success
Exp. 1	179	--	.045	-0.15	.518
Exp. 2	31, 26	2.24	.029	0.588	.583
Exp. 3	43, 50	2.11	.038	0.435	.544
Exp. 4	75, 70	2.20	.029	0.364	.585
Exp. 4	88, 91	2.06	.041	0.307	.532
P_{TES}					.051

Gervais & Norenzayan (2012) reported that each of the studies produced the pattern of results that support their theoretical stance. If the theory is correct, and the population effects are as estimated by the samples, then the probability of getting studies like these to all produce the desired pattern is the product of the success probabilities: $P_{TES} = .051$. Since

this value is smaller than the .1 criterion, readers should be skeptical about the reported experimental results as they relate to the theory.

19. Analysis of Seeley, Visscher, Schlegel, Hogan, Franks & Marshall (2012) "Stop Signals Provide Cross Inhibition in Collective Decision-Making by Honeybee Swarms"

Seeley *et al.* (2012) reported four experiments purporting to show that inhibitory stop signals influenced decision-making for a swarm of bees. Table S18 summarizes the statistics that contributed to the TES analysis.

All of the experiments investigated how a stop signal generated by some bees influenced the waggle dance being produced by other swam members. The test statistic for every experiment is a χ^2 value. Success probability was calculated with an on-line applet (Lenth, 2009). Some experiments included other tests, but they are not likely to change the TES analysis by much.

Seeley *et al.* (2012) reported that each of the studies produced the pattern of results that support their theoretical stance. If the theory is correct, and the population effects are as estimated by the samples, then the probability of getting studies like these to all produce the desired pattern is the product of the success probabilities: $P_{TES} = .957$. Since this value is larger than the .1 criterion, the TES analysis does not suggest that readers should be skeptical about the reported experimental results as they relate to the theory.

Table S18: Statistical properties of the Seeley *et al.* (2012) experiments.

	n	Test Statistic	p	Effect Size	Probability of Success
Exp. 1	109	21.90	<.0001	--	.997
Exp. 2	358	58.00	<.0001	--	1.00
Exp. 3	60	16.18	<.0001	--	.980
Exp. 4	112	16.03	<.0001	--	.980
P_{TES}					.957

20. Analysis of Shah, Mullainathan & Shafir (2012) "Some Consequences of Having Too Little"

Shah *et al.* (2012) reported five experiments purporting to show that scarcity modifies attentional allocation and explains behaviours such as overborrowing. Table S19 summarizes the statistics that contributed to the TES analysis.

In Experiment 1, participants played a wheel of fortune type game, but poor participants had fewer possible guesses than rich participants per round. After playing the game, participants completed a measure of cognitive fatigue, and poor participants showed higher fatigue than rich participants. The test statistic in Table S19 is an *F* value.

Experiment 2 explored behaviour in a game, similar to Angry Birds, that varied how rich or poor participants borrowed from future rounds (in terms of number of shots). The analysis included six hypothesis tests, which sometimes used the same datasets. One test

was for a correlation between the time spent aiming shots and subsequent borrowing among poor participants. The effect size in Table S19 is an *r* value. The success probability of this test sets an upper limit on the joint success probability of all the tests.

Experiment 3 had participants play a Family Feud type of game with rich and poor budgets that varied in the time available to answer questions. In addition, participants were assigned to conditions that prohibited borrowing time from future questions or allowed borrowing without interest or with interest. The dependent measure was performance in the game. ANOVAs reported a significant effect of borrowing conditions for the poor but a non-significant effect for the rich. The joint success probability of these findings was estimated with simulated experiments.

Experiment 4 was similar to Experiment 3, but cast the borrowing as debt that was taken out of future payments during the game. The analysis included several connected hypothesis tests, and one finding was that the poor participants performed better when they could not borrow compared to poor participants who could borrow. The estimated success probability for this result sets an upper limit for the joint success probability of the multiple tests. The test statistic in Table S19 is an *F* value.

Experiment 5 reported that poor participants were unable to take advantage of cues about topics of future questions in a Family Feud game, but that rich participants could use such cues. The *F* value in Table S19 is for the interaction of rich/poor and future views being present or absent.

Table S19: Statistical properties of the Shah *et al.* (2012) experiments.

	**n**	**Test Statistic**	**p**	**Effect Size**	**Probability of Success**
Exp. 1	28, 28	4.16	.046	0.538	.506
Exp. 2	40	--	.032	0.340	.576
Exp. 3	23, 24, 24, 24, 24, 24	Multiple tests	--	--	.618
Exp. 4	57, 57	12.81	<.001	0.666	.941
Exp. 5	68, 69	4.29	.040	0.352	.534
P_{TES}					.091

Shah *et al.* (2012) reported that each of the studies produced the pattern of results that support their theoretical stance. If the theory is correct, and the population effects are as estimated by the samples, then the probability of getting studies like these to all produce the desired pattern is the product of the success probabilities: $P_{TES} = .091$. Since this value is smaller than the .1 criterion, readers should be skeptical about the reported experimental results as they relate to the theory.

References not listed in main article

Aviezer, H., Trope, Y. & Todorov, A. (2012). Body cues, not facial expressions, discriminate between intense positive and negative emotions. *Science*, *338*, 1225-1229.

Chapman, H. A., Kim, D. A., Susskind, J. M. & Anderson, A. K. (2009). In bad taste: Evidence for the oral origins of moral disgust. *Science*, *323*, 1222-1226.

Duncan, K., Sadanand, A. & Davachi, L. (2012). Memory's penumbra: Episodic memory decisions induce lingering mnemonic biases. *Science*, *337*, 485-487.

Faul, F., Erdfelder, E., Lang, A-G., & Buchner, A. (2007). G*power 3: A flexible statistical power analysis program for the social, behavioral, and biomedical sciences. *Behavior Research Methods*, *39*, 175-191.

Fayard, J. V., Bassi, A. K., Bernstein, D. M. & Roberts, B. W. (2009). Is cleanliness next to godliness? Dispelling old wives' tales: Failure to replicate Zhong and Liljenquist (2006). *Journal of Articles in Support of the Null Hypothesis*, *6*, 21-28.

Hedges, L. V. (1981). Distribution theory for Glass's estimator of effect size and related estimators. *Journal of Educational Statistics*, *6*, 107-128.

Koriat, A. (2012). When are two heads better than one and why? *Science*, *336*, 360-362.

Lenth, R. V. (2009). Java applets for power and sample size [Computer software]. Retrieved June 22, 2013, from http://www.stat.uiowa.edu/~rlenth/Power.

Maya-Ventencourt, J. F., Sale, A., Viegi, A., Baroncelli, L., De Pasquale, R., O'Leary, O. F., Castrén, E. & Maffei, L. (2008). The antidepressant fluoxetine restores plasticity in the adult visual cortex. *Science*, *320*, 385-388.

Mulcahy, N. J. & Call, J. (2006). Apes save tools for future use. *Science*, *312*, 1038-1040.

R Development Core Team. (2013) *R: A language and environment for statistical computing*. R Foundation for Statistical Computing, Vienna, Austria. http://www.R-project.org/.

Sparrow, B., Liu, J. & Wegner, D. M. (2011). Google effects on memory: Cognitive consequences of having information at our fingertips. *Science*, *333*, 776-778.

Thomsen, L., Frankenhuis, W. E., Ingold-Smith, M. & Carey, S. (2011). Big and mighty: Preverbal infants mentally represent social dominance. *Science*, *331*, 477-480.

Wood, J. N. & Hauser, M. D. (2011). Replication of "The Perception of Rational, Goal-Directed Action in Nonhuman Primates". *Science*, DOI: 10.1126/science.1202596.
