# Supplementary figures and images for: Excess Success for Psychology Articles in the Journal Science
Source: PLoS One. 2014 Dec 4;9(12):e114255. doi: 10.1371/journal.pone.0114255 (PMC4256411; doi:10.1371/journal.pone.0114255)

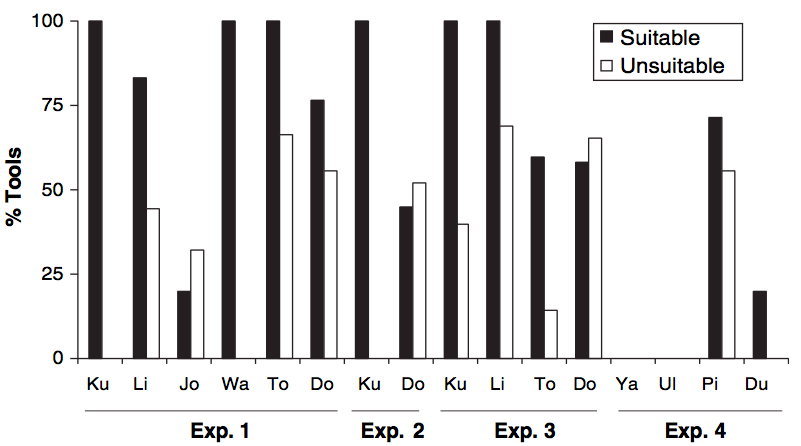

Supplement: Information S2 — TES analysis calculations. This compressed file contains a directory for every article in Table 2. Each directory includes a text file describing the location of the statistics taken from the article that were used for the TES analysis. It also includes a spreadsheet that summarizes the statistics, computes effect sizes (where appropriate), and lists the estimated success probability for each experiment. The directory also includes any R source code that was used to estimate success probability. (ZIP) [file pone.0114255.s002.zip › TESAnalyses/CannotComputePower/MulcahyCall/Simulations/Screen Shot 2013-10-02 at 3.14.43 PM.png]
